# Impact of early career medical research funding on subsequent publication outcomes and research income for female and male applicants in the United Kingdom

**DOI:** 10.1038/s41467-026-73009-z

**Published:** 2026-06-23

**Authors:** Charitini Stavropoulou, Ian Viney

**Affiliations:** 1 City St George’s, University of London, London, UK; 2 UK Research and Innovation, London, UK

**Keywords:** Scientific community, Social sciences

## Abstract

The current debate in science funding of early-career researchers is shifting from exploring its effects on future outcomes to understanding who is more likely to benefit from it. In this paper, we explore the effect of research funding among male and female applicants. Using propensity score weighting on data from the Medical Research Council in the United Kingdom, we examine the effect on research income, scientific productivity (publications), as well as scientific influence (citations, relative citation ratio, field citation ratio and Altmetrics score). Successful applicants in both groups secure more future research funding than unsuccessful ones. In addition, successful female applicants receive more citations and are better cited in their field than female applicants who did not receive funding. There is no statistically significant difference in scientific productivity or influence between successful and unsuccessful male applicants.

## Abstract (web summary)

The authors examine the impact of funding for future publishing outcomes and research income of female and male early career researchers based on data from the Medical Research Council in the United Kingdom

## Introduction

Securing funding in the early stages of one’s research career is crucial. Not only does it determine whether a researcher can develop their own project and build their team, but it can also define whether they secure a permanent academic job at the university or leave academia. With precarious jobs among early career researchers becoming the norm increasingly in higher education globally, finding ways to retain talent is a concern shared among research institutions, policy officials and funders^1^.

Funding bodies around the world have established early career schemes aiming to support post-doctoral researchers to pursue their own research agenda and make the transition to research independence^2,3^. Schemes like the Pathway to Independence Awards from the National Institutes for Health in the US^2^, the Starting Grants from the European Research Council^3^ and early career awards from charity and public funders in the UK, allow early career scholars to compete with peers at a similar stage and increase their chances of success. Empirical evidence on the effect that early career schemes have on individual researchers is scarce and offers contradictory results on whether these schemes support individuals in accumulating more research funding in the future and improving scientific outcomes^4,5^.

Yet, the more recent debate on scientific funding is now shifting towards exploring not only what the effect of early career funding on the quality and quantity of scientific outputs is, but also who is more likely to benefit from such support^6,7^. Gender has been at the centre of this debate, not least because of a growing literature identifying gender gaps in securing research funding. Female scientists are underrepresented in major funding schemes^8^, are more poorly evaluated than male counterparts in peer review processes^9–11^ and are less likely to be successful in being awarded research grants than men^11,12^. Yet, despite clear evidence on the challenges of female researchers securing science funding, very little is known about what happens to them *after* they receive a grant.

In this work, we explore the impact that funding of early career researchers (ECR), defined as those applying to secure their first grant as Principal Investigators (PI), has on researchers’ subsequent academic performance, focusing on whether the effect may be different for male and female researchers. To do so, we use a rich dataset including both successful and unsuccessful candidates who applied for an early career scheme from the Medical Research Council (MRC) in the UK between 2006 and 2016. We focus the main analysis on those individuals who reached the board level (last stage) of the application process and use a propensity score weighting method, first for the full sample and then separately for male and female candidates. We know, of course, that successful candidates are not selected at random. Our model balances any differences between the successful and unsuccessful groups in observable characteristics at the stage of submission, which allows for the differences in future outcomes to be attributed to the grant received. We have a number of observable characteristics that previous literature has shown to matter in securing funding: ethnicity, age, academic institution, clinical background, previous applications, publication and citation metrics and previous research income^4–6,9^. Very importantly for our analysis, we have information on the applicant’s sex, as self-reported by them on their application. As outcome measures, we consider the publication and citation metrics as well as income generated post-submission (see methods section for more details). We go beyond research income and scientific productivity and examine scientific influence, including metrics such as the Relative Citation Ratio (RCR), the Field Citation Ratio (FCR) and the Altmetrics score^13^.

## Results

### Descriptive statistics

The main characteristics of the full sample of 2,030 who applied for an early career scheme at the MRC between 2006 and 2016 can be found in Table 1.

**Table 1:** Descriptive characteristics of the full sample

	UNSUCCESSFUL	SUCCESSFUL	TOTAL
**Scheme**			
CDA	620	107	727
CSF	286	86	372
NIRG	716	215	931
**Sex**			
Female	692	154	846
Male	902	252	1154
**Ethnicity**			
White	1256	340	1596
Non-White	276	53	329
**Institution in London/Oxbridge**			
No	847	198	1045
Yes	775	210	985
**Average age**	36.804	36.248	36.69
**TOTAL**	1622	408	2030

Career Development Award (CDA), Clinician Scientist Fellowship (CSF), New Investigator Research Grant (NIRG). Missing values were omitted for illustration purposes.

Of the full sample, 1171 applicants were declined at triage and 859 arrived at the board. From those arriving at the board, 451 were unsuccessful and 408 received an award (Table 2). Our analysis focuses only on those applicants who have reached the board. We further excluded those applicants who were unsuccessful from the MRC, but secured an early career award from another funding body (*n* = 45). That leaves a clean sample of 814 applicants, of whom 408 were successful. Table 3 shows the success rates by the applicant’s sex, first for the full sample and then for the analysis sample (only those who reached the board level).

**Table 2:** Selection process

	Declined at triage	Arrived at board	Total
**Unsuccessful**	1171	451	1622
Female	511	181	692
Male	640	262	902
**Successful**	0	408	408
Female	0	154	154
Male	0	252	252
Total	1171	859	2030

Missing values were omitted for illustration purposes.

**Table 3:** Success rate by sex

	Successful	Unsuccessful	Success rate
**Full Sample**			
Female	154	692	18.2%
Male	252	902	21.8%
**At board**			
Female	154	181	46%
Male	252	262	49%

Missing values were omitted for illustration purposes.

### Baseline comparisons

First, we run a logistic regression of the probability of success on observable characteristics at submission, both for the total sample (Table 4) and then separately for female and male applicants (Table 5). When considering the total sample in Table 4, we show that there is no statistically significant difference in the probability of success for male and female applicants when controlling for a number of observable characteristics. Results also show that there is no statistically significant difference between successful and unsuccessful applicants at submission in terms of the applicant’s ethnic background (OR = 0.923, 95% CI [0.570, 1.494], *p* = 0.743) or the research institution they come from (OR = 1.157, 95% CI [0.834, 1.606], *p* = 0.382). They do not statistically significantly differ in terms of publications (OR = 0.913, 95% CI [0.710, 1.175], *p* = 0.479), citations (OR = 1.068, 95% CI [0.788, 1.447], *p* = 0.671), relative citation ratio (OR = 1.014, 95% CI [0.680, 1.515], *p* = 0.944), or field citation ratio before submission (OR = 1.107, 95% CI [0.772, 1.587], *p* = 0.581.). However, they statistically significantly differ in terms of age with successful applicants being younger (OR = 0.918, 95% CI [0.880, 0.956], *p* < 0.001); whether they had secured funding prior to the application (OR = 2.145, 95% CI [1.480, 3.110], *p* < 0.001), whether they had applied in the past for one of these schemes (OR = 1.870, 95% CI [1.275, 2.743], *p* = 0.001) and whether they were clinical academics (OR = 0.617, 95% CI [0.407, 0.934], *p* = 0.022). They also differ statistically significantly in their average Altmetrics prior to submission (OR = 0.732, 95% CI [0.581, 0.922], *p* = 0.008). We observe similar findings for the total sample, as well as the female and male applicants separately (Table 5). Tables 4 and 5 also illustrate that after applying propensity score weighting, these differences are balanced out both for the total sample and the subgroup analysis.

**Table 4:** Differences in observable characteristics between successful and unsuccessful applicants in the total sample before and after propensity score weight adjustment at submission, using multivariable logistic regression

	UNWEIGHTED						WEIGHTED					
	Odds ratio	Standard Error	Z	p value	CI 95%		Odds ratio	Standard Error	Z	p value	95% CI	
**TOTAL SAMPLE**												
Age	0.918	0.019	−4.060	<0.001	0.880	0.956	1.004	0.021	0.180	0.857	0.963	1.046
Sex	1.124	0.196	0.670	0.501	0.799	1.581	1.016	0.178	0.090	0.929	0.720	1.433
Ethnicity	0.923	0.227	−0.330	0.743	0.570	1.494	0.921	0.228	−0.330	0.738	0.567	1.495
Institution in London/Oxbridge	1.157	0.194	0.870	0.382	0.834	1.606	1.031	0.174	0.180	0.856	0.741	1.435
Publications (standardised)	0.913	0.117	−0.710	0.479	0.710	1.175	1.010	0.131	0.080	0.939	0.783	1.302
Citations (standardised)	1.068	0.166	0.430	0.671	0.788	1.447	0.947	0.146	−0.350	0.723	0.699	1.282
RCR (standardised)	1.014	0.207	0.070	0.944	0.680	1.515	1.040	0.216	0.190	0.852	0.692	1.561
FCR (standardised)	1.107	0.204	0.550	0.581	0.772	1.587	1.043	0.192	0.230	0.818	0.727	1.496
Altmetrics (standardised)	0.732	0.086	−2.650	0.008	0.581	0.922	0.935	0.086	−0.740	0.461	0.781	1.119
Previous Income (binary)	2.145	0.406	4.030	<0.001	1.480	3.110	1.002	0.191	0.010	0.991	0.689	1.457
Past applications (binary)	1.870	0.365	3.210	0.001	1.275	2.743	1.043	0.203	0.220	0.829	0.712	1.527
Clinical background	0.617	0.131	−2.280	0.022	0.407	0.934	1.012	0.216	0.060	0.954	0.666	1.538

p-values based on logistic regressions analysing those applicants who were considered at the board. Statistically significant results highlighted in bold. *CI* Confidence Interval, *RCR*Relative Citation Ratio, *FCR* Field Citation Ratio.

**Table 5:** Differences in observable characteristics between successful and unsuccessful male and female applicants before and after propensity score weight adjustment at submission, using multivariable logistic regression

	UNWEIGHTED						WEIGHTED					
	Odds ratio	Stand Error	Z	p value	CI 95%		Odds ratio	Standard Error	Z	p value	95% CI	
**MALE ONLY**												
Age	0.866	0.027	−4.580	<0.001	0.814	0.921	0.999	0.031	−0.030	0.978	0.941	1.061
Ethnicity	1.055	0.318	0.180	0.860	0.584	1.905	0.928	0.284	−0.240	0.808	0.510	1.690
Institution in London/Oxbridge	1.185	0.260	0.770	0.439	0.771	1.821	0.991	0.221	−0.040	0.966	0.640	1.533
Publications (standardised)	0.806	0.128	−1.360	0.173	0.590	1.099	0.998	0.153	−0.010	0.989	0.738	1.349
Citations (standardised)	1.210	0.248	0.930	0.354	0.809	1.809	0.994	0.208	−0.030	0.975	0.659	1.498
RCR (standardised)	1.135	0.279	0.510	0.607	0.701	1.837	0.993	0.251	−0.030	0.979	0.605	1.630
FCR (standardised)	0.867	0.208	−0.590	0.553	0.542	1.388	1.064	0.260	0.260	0.798	0.659	1.718
Altmetrics (standardised)	0.701	0.122	−2.040	0.041	0.498	0.986	0.925	0.125	−0.580	0.565	0.709	1.207
Previous Income (binary)	2.403	0.599	3.520	<0.001	1.474	3.916	0.961	0.243	−0.160	0.875	0.585	1.579
Past applications (binary)	1.830	0.479	2.310	0.021	1.095	3.058	1.072	0.279	0.270	0.788	0.644	1.786
Clinical background	0.719	0.186	−1.270	0.203	0.433	1.194	1.067	0.284	0.240	0.808	0.634	1.796
**FEMALE ONLY**												
Age	0.975	0.029	−0.860	0.391	0.920	1.033	1.006	0.029	0.210	0.830	0.950	1.066
Ethnicity	0.689	0.310	−0.830	0.408	0.285	1.664	0.898	0.399	−0.240	0.808	0.376	2.145
Institution in London/Oxbridge	1.150	0.308	0.520	0.600	0.681	1.944	1.025	0.275	0.090	0.927	0.606	1.735
Publications (standardised)	1.251	0.317	0.880	0.376	0.762	2.055	0.907	0.219	−0.410	0.685	0.564	1.456
Citations (standardised)	0.881	0.225	−0.500	0.619	0.533	1.454	1.013	0.252	0.050	0.960	0.621	1.650
RCR (standardised)	0.912	0.377	−0.220	0.824	0.406	2.051	0.964	0.399	−0.090	0.929	0.428	2.168
FCR (standardised)	1.552	0.477	1.430	0.152	0.850	2.834	1.078	0.335	0.240	0.810	0.586	1.983
Altmetrics (standardised)	0.727	0.122	−1.900	0.057	0.523	1.009	0.918	0.116	−0.680	0.498	0.716	1.176
Previous Income (binary)	1.866	0.571	2.040	0.041	1.024	3.399	1.038	0.317	0.120	0.903	0.570	1.889
Past applications (binary)	2.023	0.621	2.300	0.022	1.108	3.690	1.001	0.308	0.000	0.996	0.548	1.832
Clinical background	0.449	0.177	−2.030	0.042	0.207	0.972	0.994	0.391	−0.020	0.987	0.460	2.148

p-values based on logistic regressions analysing those applicants who were considered at the board. Statistically significant results highlighted in bold. *CI* Confidence Interval, *RCR* Relative Citation Ratio, *FCR* Field Citation Ratio.

### Overall impact of the award on publication outcomes and research income

Table 6 presents the effect of the award on the number of publications per year, the number of publications as last author per year, citations per year, average RCR, average FCR and Altmetrics score. It also presents the effect of the award on generating additional research income post-submission. We find no statistically significant differences between the successful and unsuccessful candidates in terms of scientific productivity, i.e. volume of publications and citations and no statistically significant effect on scientific influence, i.e. citation ratios or Altmetrics. Instead, there is a statistically significant effect of the award on research income, both in terms of total annual income (ATE = 3.224, *p* < 0.001, Cohen’s d = 0.599) and income as PI (ATE = 6.600, *p* < 0.001, Cohen’s d = 1.233).

**Table 6:** Effect of award on publications, citations and research income using propensity score weighting

	Scientific productivity	Scientific Influence				Research Income	
	Publications per year	Citations per year	Average RCR	Average FCR	Average Altmetrics	Total per year	PI income per year
ATE	−0.001	0.033	−0.039	−0.020	−0.024	**3.224**	**6.600**
Standard error	0.070	0.066	0.098	0.085	0.097	**0.416**	**0.345**
P value	0.983	0.619	0.693	0.811	0.808	**<0.001**	**<0.001**
95% CI	−0.139, 0.136	−0.097, 0.162	−0.231, 0.154	−0.187, 0.146	−0.214, 0.166	**2.428, 4.060**	**5.924, 7.276**
Cohen’s d	−0.001	0.032	−0.038	−0.020	−0.025	**0.599**	**1.233**

*ATE* Average treatment effects, *CI* Confidence Interval, *RCR* Relative Citation Ratio, *FCR* Field Citation Ratio, *PI* Principal Investigator

(statistically significant results highlighted in bold).

### Subgroup analysis by sex

**Fig. 1:**
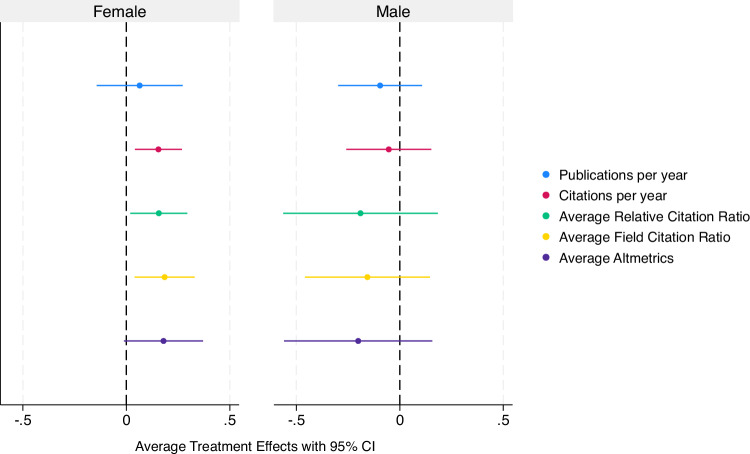
Effect of award on scientific productivity and influence. Effect of award by sex using propensity score weighting (female *n* = 264; male *n* = 395). (*All AverageTreatment Effects and their standard errors are presented on* Table 5).

Table 7 presents the results on the effect of an award on publications and citations, using propensity score weighting considering other observable characteristics at the time of submission. Here we observe a clear sex difference in the impact of an award on citations in the years following the submission; while for male applicants the results are similar to the overall sample, when the analysis is done on the female group only, successful applicants are cited more (ATE = 0.155, *p* = 0.007, cohen’s d = 0.262), have a higher relative citation ratio (ATE = 0.157, *p* = 0.026, cohen’s d = 0.245) and a higher field citation ratio (ATE = 0.185, *p* = 0.013, cohen’s d = 0.267). Figure 1 summarises the findings graphically.

**Table 7:** Subgroup analysis of the effect of the award on publications and citations using propensity score weighting

	Scientific productivity	Scientific Influence			
	Publications per year	Citations per year	Average RCR	Average FCR	Average Altmetrics
FEMALE ONLY					
ATE	0.064	**0.155**	**0.157**	**0.185**	0.180
Standard error	0.106	**0.058**	**0.070**	**0.074**	0.098
P value	0.544	**0.007**	**0.026**	**0.013**	0.066
95% CI	−0.143, 0.272	**0.042, 0.268**	**0.019, 0.295**	**0.040, 0.330**	−0.012, 0.371
Cohen’s d	0.074	**0.262**	**0.245**	**0.267**	0.213
MALE ONLY					
ATE	−0.095	−0.053	−0.190	−0.157	−0.201
Standard error	0.103	0.105	0.191	0.154	0.183
P value	0.357	0.612	0.319	0.309	0.271
95% CI	−0.298, 0.107	−0.259, 0.152	−0.564, 0.183	−0.458, 0.145	−0.560, 0.157
Cohen’s d	−0.088	−0.044	−0.158	−0.132	−0.204

*ATE* Average treatment effects, *CI* Confidence Interval, *RCR* Relative Citation Ratio, *FCR* Field Citation Ratio.

(statistically significant results highlighted in bold).

When focusing on research income generated post submission, there is a significant positive effect of the award on all groups, both in terms of total income as well as income as PI (Table 8).

**Table 8:** Subgroup analysis of effect of award on research income using propensity score weighting

	Research Income	
	Total per year	PI income per year
FEMALE ONLY		
ATE	**4.098**	**7.422**
Standard error	**0.647**	**0.518**
P value	**<0.001**	**<0.001**
95% CI	**2.830, 5.366**	**6.407, 8.438**
Cohen’s d	**0.726**	**1.333**
MALE ONLY		
ATE	**2.886**	**6.165**
Standard error	**0.520**	**0.463**
P value	**<0.001**	**<0.001**
95% CI	**1.867, 3.906**	**5.258, 7.072**
Cohen’s d	**0.552**	**1.191**

*ATE* Average treatment effects, *CI* Confidence Interval, *PI* Principal Investigator.

(statistically significant results highlighted in bold).

### Interviews with award holders

Interviews with successful award holders, part of a bigger qualitative study ^14^, offered interesting insights into our findings. They are based on interviews with successful candidates from the main sample of the quantitative analysis presented above (*N* = 46), with additional interviews conducted with five researchers from a comparable scheme to assess the broader applicability of the themes.

Although work-life balance was a topic mentioned by both male and female researchers, the qualitative analysis revealed significant differences, with 14 out of 24 female interviewees having taken maternity leave during their award, while no male interviewee mentioned a career break for family reasons. Women talked about the challenges they face in academia, but highlighted the importance of the protected time that the MRC early career schemes offered to them and praised the maternity policies that were in place. They also referred to leadership schemes tailored specifically to women in academia and the mentorship support they received as part of their fellowships and awards. This additional support that is offered to women may partly explain why they benefit more than men receiving these fellowships.

## Discussion

Our findings contribute to a limited literature on the effect of science funding on early-career researchers. In addition to exploring the effect on income generation, our analysis also considers the effect separately for male and female applicants and it goes beyond productivity metrics to include measures of citation outreach and scientific influence within a researcher’s field.

In the overall sample, we find evidence of a Matthew effect similar to that of Bol et al.^4^; receiving an early career grant results in further research income being generated post submission, but no other differences in academic outputs. Yet, we find strong evidence that successful female researchers improve their citations, citation outreach and influence in their field more than those who did not win an award.

Prior literature has focused on disparities in grant submission and success rates, showing that fewer women apply for grants and receive smaller grants than men^7,10,12^. Our study found no statistically significant difference between female and male candidates in receiving an award or fellowship, but we find clear evidence that women who win an award increase their research outreach and impact in comparison to women who do not. The results are significant in light of recent evidence showing that women are credited less in science than men^15^ and show that receiving research funding can help women stand out in their research field.

Our findings on publications are consistent with previous evidence that securing a research grant does not necessarily improve scientific productivity as captured by academic publications. Using data from researchers in different career stages applying for National Institutes of Health (NIH) research grants, Jacob and Lefgren^16^ showed that receipt of a research grant (worth roughly $1.7 million) led to only one additional publication over the next five years. Yet, this is the first analysis on early career researchers that includes publication metrics that go beyond productivity, i.e. number of publications and citations, but also variables that reflect the candidate’s relative performance in comparison to others in the field. This is important as new research suggests that moving towards scientific influence may be more important than scientific productivity^17^ and this is particularly important for early career researchers who are trying to establish a reputation as an independent researcher in their field.

Equality, Diversity and Inclusion (EDI) policy interventions in funding schemes can create fairer systems. During Covid-19, the Canadian Institutes of Health Research (CIHR) offered compensation for dependent caregiver costs, more generous parental leave credits and extended early career status; an intervention that led to more applications and success rates among women^18^. More work could shed further light on what explains the positive effect we observe among women, but our findings suggest that policy interventions post-award are beneficial to female ECRs. The UKRI published an EDI strategy and action plans to promote ‘a more diverse and inclusive research and innovation system’^19^.

Our study is not without limitations. First, it considers a small and specific sample of ECRs who have received funding from a UK funder of medical research. Larger studies looking across fields would be helpful to consider the role of sex and other characteristics, such as ethnicity, when analysing the effects of public funding on individual researchers. Second, the MRC is a major funding body of medical research in the UK, but not the only one. The Wellcome Trust and the National Institute for Health and Care Research (NIHR) offer alternative schemes for early-career researchers, for which we have no information. To the extent that we could control for the fact that we only analyzed MRC applicants, we excluded from our database applicants who appeared as unsuccessful in our data but had successfully received another fellowship from another funder. Third, defining success in terms of scientific productivity, outreach and research income generation is not necessarily the only goal of an ECR award or fellowship. They are nonetheless important metrics upon which academics are often evaluated and promoted and our attempt to go beyond crude numbers, e.g. number of publications, aims to offer a broader perspective. Having information on career progression, such as tenure and promotions, would have been useful to see how these awards shape individuals’ development. Finally, we are aware that the analysis is based on sex rather than gender, as this was the information collected during the period we explored. We would welcome further explorations in this area that go beyond a binary defined variable.

Our study finds within-group differences when looking at female and male applicants separately. The effect is mostly on scientific influence and academic outreach and has significant implications for female researchers who often face more challenges in academic progression. An important future direction in this area would be an extension of this analysis to a wider group that consists of different disciplines, as well as an exploration of similar patterns in different ethnic groups.

## Methods

The study received ethical approval from the School of Health Sciences (SHS) at City, University of London, Ethics Committee (ETH2021-1239 both for the quantitative component and the qualitative interviews).

### Empirical context of the study: the MRC’s early career schemes

The MRC is the largest public funding body of medical research in the UK and one of the largest funders of medical research in the world^20^. Since 2019, it has been one of the nine councils that form the UK Research and Innovation (UKRI), funded through the science budget of the UK’s Department for Business, Energy and Industrial Strategy.

MRC’s annual investment in support for early career researchers is approximately £30 million (four per cent of the portfolio). The MRC’s early career schemes include two fellowship programmes, namely the Career Development Award (CDA) and Clinician Scientist Fellowships (CSF) and one grant scheme that is aimed at early career researchers only: the New Investigator Research Grant (NIRG). These are the schemes we consider in this study. These are all individual awards given to the researchers who submit a proposal on a topic they have started developing an expertise in and need funding to establish their own lab or team to develop it further. All three schemes are aimed at applicants with a PhD who provide clear evidence of being on the trajectory to become independent researchers in their field and have not received previous income as a PI. Yet, there are differences among the three awards. Applications for a CSF are available only to healthcare professionals, while the two fellowship schemes (CSF and CDA) come with a tailored training program for the professional development of the individual fellows, which NIRGs' awards do not cover. The duration of the award and hence the amount requested is not the same, depending on the costs associated with running a lab or a team, but the schemes do have a list of elements they do not cover, such as funding for PhD studentships.

Applicants are requested to submit a research proposal and a letter of support from a UK institution that is willing to host the researcher if they are successful. The applications are then peer-reviewed externally by researchers in relevant fields and are given a score, which determines whether their application will be considered at a board level. The panel board decides on which applicants will be funded.

### Data and empirical strategy

#### Sample

Our sample consists of every individual researcher who applied for an MRC early-career fellowship or award between 2006 and 2016. This allows us to follow individual researchers for at least 5 years post-submission, as we hold data on external funding and publications up until 2021. For the purposes of the main analysis, we focus only on those applicants who were assessed at the board level. Furthermore, we exclude applicants who appear as unsuccessful in our dataset but secured an early career award from another funding body, such as the Wellcome Trust or the National Institute for Health and Care Research (NIHR).

#### Datasets and variables

We match three databases: (1) the MRC’s own records for key baseline characteristics (SIEBEL); (2) data sourced from Dimensions, an inter-linked research information system provided by Digital Science (https://www.dimensions.ai); and (3) manually identified information from LinkedIn and Google for career progression.

More specifically, the MRC holds information on the applicants’ characteristics at the time of submission. These include the applicant’s sex (female or male), ethnicity (white or non-white), age at submission (in years) and hosting research organisation. We classify the hosting research organisations into two categories: those belonging to Universities based in London, Oxford or Cambridge and those outside that region. For each applicant, we also know the year of submission, the scheme they applied for, whether clinical or non-clinical academics, whether they had applied again in the past, whether they made it to the board or were declined at triage and whether they were successful or not. Informed consent was not required for this study, as the analysis used secondary data collected and processed by UKRI under its established terms and conditions and statutory functions (https://www.ukri.org/publications/terms-and-conditions-for-research-grants/.

Data on external funding and publications are obtained from Dimensions. For each individual in our sample, Dimensions provides data on grants that researchers secure per calendar year, which are split into total income and income as PI. On the basis of these variables, we calculate the cumulative income secured by each applicant over the years.

For publication outcomes, using Dimensions, we are able to calculate both measures of scientific productivity and scientific influence. For scientific productivity, we have information on the total number of publications each applicant had prior to submission of their application to the MRC and for each year post-submission. We retrieve the number of publications that individuals published as senior (last) authors each year post-submission. In addition, we have information on scientific influence. More specifically, for every paper we know the number of citations, the Relative Citation Ratio (RCR), a weighted measure of the number of citations a paper receives in comparison to other papers in the same field^21^, their Field Citation Ratio (FCR), which divides the total number of times a paper has been cited by the average number received by documents published in the same year in the same field^13^ and the Altmetrics score, a weighted count of the attention an article has received^13^. Based on these citation metrics, we calculate the average RCR, average FCR and average Altmetrics score each individual received for the papers they published after submission of an application to the MRC. Dimensions also provide information on the field of research each paper is published in. For each individual, we identify the field they most frequently publish in.

Finally, from Dimensions, we identify the research institute each applicant is currently in, using the affiliation they have in their latest publication as a proxy. Dimensions was chosen, given an ongoing collaboration they had with the MRC at the time of the analysis. It has been shown to perform as well as Scopus and Web of Science in citation tracking^22^. As mentioned earlier, one possible noise in our data is the fact that some candidates who appear as unsuccessful in our data may have received an early career fellowship or award from another funding body. Therefore, we search all the unsuccessful candidates who appeared as PIs in our database and identify those who had received an early career award from another body by looking into Google, their personal websites or LinkedIn.

Table S1 in the Supplementary Information File summarises the variables we use from the three databases.

### Empirical strategy

We use propensity score weighting for our analysis^23^. The analysis accounts for differences observed at the baseline (submission year) between the treatment (successful) and control group (unsuccessful) and by balancing these differences, it allows for the differences in outcomes observed in the future to be attributed to the treatment (grant received).

**Fig. 2:**
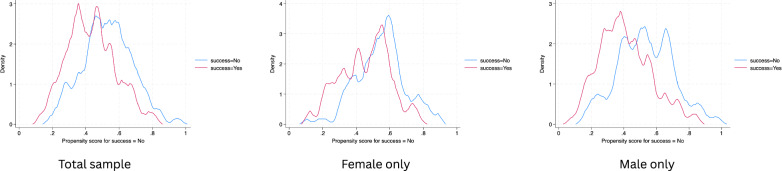
Post estimation overlap plots. Overlap plots between successful and unsuccessful groups for total, female and male samples separately.

For the results of the analysis to be valid, a number of assumptions need to hold^24^. First, unconfoundness or conditional exchangeability requires that all variables that affect the likelihood of receiving a grant are observed. Although this assumption cannot be tested empirically, we have rich information on pre-treatment outcomes, suggesting that the assumption that all relevant variables are observed may be a reasonable approximation^25^. Second, the positivity or overlap assumption requires that each individual has a positive probability of receiving each treatment. Unlike the other assumptions, the overlap assumption can be tested empirically, using a postestimation command that plots the estimated densities of the probability of getting each treatment level. We present these plots in Fig. 2 for the full sample, the female and the male groups. The estimated densities of successful and unsuccessful applicants have most of their respective masses in regions in which they overlap each other and they are not near 0 or 1. Thus, there is no evidence that the overlap assumption is violated. Third, the stable unit treatment value assumption (SUTVA) states that for a given individual, the treatment of another individual does not affect the outcome of the treatment received by that individual. We have no reason to assume there are any spillover effects that may violate the stability assumption. Fourth, the consistency assumption requires that the treatment is sufficiently well defined and is violated if there are multiple different versions of the treatment. In our analysis, we consider three different schemes that support early career researchers. For the overall analysis, the estimated effect will be an average of these different effects.

We estimate the average treatment effect that the award has on publication outcomes and generating external funding, by conditioning on the probability of receiving an award on observed covariates (propensity score). We use an inverse probability score, meaning that applicants who received an award get a weight of 1 and those unsuccessful get a weight of *P*(*T*) / (1 − *P*(*T*)). This weighting allows the two groups of successful and unsuccessful candidates to be similar across a number of observed characteristics at the baseline, namely the time of submission of an application to the MRC. Hence, the differences post-submissions will be attributed to the effect of the award. We run balanced diagnostics to confirm whether the propensity score model is appropriately specified (Table S2 in the Supplementary File).

Previous studies in the area of ECR funding had used regression discontinuity methods^4,5^. This type of analysis requires information on the scores ECR applicants received to explore differences between successful and unsuccessful candidates around the threshold. Our data on scores was incomplete and did not allow this type of analysis. We believe the propensity score weighting offers the most appropriate approach to address the paper’s aim.

Our data allow us to consider a number of observed covariates that determine the probability of receiving an award: sex, age, ethnic background, the hosting institution, whether they are clinical academics or not, the applicant’s citation and publications at submission, whether they had received research funding in the past or not and whether they had applied again in the past for an MRC fellowship or award. To allow for differences in citation and publication cultures in different schemes, we calculate the z-scores of these variables for each applicant within their own field, similar to Bol et al^3^.

Our outcome variables include three main areas. First, research income per year post-submission in total and as PI. Second, scientific productivity, including the average number of publications per year, the average number of publications per year as first author and the average number of citations per year. Third, scientific influence as measured by the Relative Citation Ratio, the Field Citation Ratio and the Altmetrics score^13^.

Effect sizes were estimated using Cohen’s d.

### Interviews with award holders

To get a better understanding of our findings, we conducted in-depth interviews with successful applicants. This was part of a larger qualitative study we conducted between April and November 2021, including 27 male and 24 female recipients of an ECR award from the MRC, which explored the challenges of navigating an early career in medical research^14^. All participants gave written consent prior to their interview. For the interviews, participants were stratified to include a balanced representation from all three schemes, both from universities concentrated around London, Oxford and Cambridge and other institutions across the country. The study included both current (at the time of the interview) and past fellows. The interviews focused on the individuals’ journey since they received the award and were conducted online, due to social distancing measures put in place during the study period. Interviews were analysed using a systems theory perspective to gain a holistic understanding of the challenges early career researchers face.

The qualitative interviews were conducted with a subset of participants from the main quantitative sample, specifically individuals who were successful candidates in the scheme under study (*N* = 46). In addition, five researchers from a separate but comparable programme (UKRI’s Future Leaders Fellowship) were included to assess whether the observed themes extended beyond the primary sample.

The larger qualitative study also included focus groups, but these were not used for the interpretation of our current quantitative analysis.

### Ethics statement

The study received ethical approval from the School of Health Sciences (SHS) at City, University of London, Ethics Committee (ETH2021-1239).

### Reporting summary

Further information on research design is available in the Nature Portfolio Reporting Summary linked to this article.

## Supplementary information

Supplementary Information File

Reporting Summary

Transparent Peer Review file

## Data Availability

This paper makes use of restricted data from UK Research and Innovation (UKRI), protected by the UK Data Protection Act 2018. This data was accessed by Dr. Stavropoulou as part of a collaborative research project with UKRI. UKRI makes unrestricted data on its funding available via https://gtr.ukri.org/. Dimensions data was obtained from https://www.dimensions.ai. We manually searched the LinkedIn dataset and Google engines. A minimum dataset, created using the datasets above and removing any identifiable information is available upon request for accredited researchers from the corresponding author. The authors do not have permission to share data from the qualitative interviews^14^ used in this study. Participants did not provide consent for their full interview transcripts to be shared. Ethical approval for this study was granted on the basis that only anonymised excerpts would be used in publications. In addition, due to the highly contextual nature of the interviews (e.g., references to specific projects, institutions and career paths), the data cannot be effectively de-identified without risking participant identification.

## Code availability

The code^26^ used to produce the results of the manuscript is available here: https://github.com/cstavropou/mrc_ecr_code/.
