# Supplementary material for: Impact of early career medical research funding on subsequent publication outcomes and research income for female and male applicants in the United Kingdom
Source: Nat Commun. 2026 Jun 23;17:7909. doi: 10.1038/s41467-026-73009-z (PMC13442836; doi:10.1038/s41467-026-73009-z)
Supplement: Supplementary file 1 [file 41467_2026_73009_MOESM1_ESM.pdf]

Supplementary Information File

Table S1. Description of variables and data sources

Variable	Description	Source
At baseline		
Sex	Applicant's sex; female (0) or male (1)	SIEBEL
Ethnicity	Applicant is from; White (0) or Non-White (1) background	SIEBEL
Age	Applicant's age at submission in years	SIEBEL
Hosting institution	Institution outside (0) or in the London/Oxford/Cambridge (1) area	SIEBEL
Scheme	Career Development Award (1), Clinician Scientist Fellow (2) or New Investigator Research Grant (3)	SIEBEL
Clinical	Whether the applicant is clinical (1) or not (0)	SIEBEL
Submission year	Year of applying for an early career scheme	SIEBEL
Success	Whether the applicant was successful (1) or not (0)	SIEBEL
Board	Whether the applicant made it to the board (1) or not (0)	SIEBEL
Past applications	Whether the applicant had applied previously to the MRC (1) or not (0)	SIEBEL
Publications	z-score of total publications per year prior to submission by field	Dimensions
Citation variables	z-score of ln 1 + total of citations prior to submission by scheme and year. 'relative citation ratio', 'relative field ratio', 'Altmetrics'.	Dimensions
Research income – pre submission	Whether the applicant has secured funding prior to submission (1) or not (0).	Dimensions
Field of Research	Field of research level 1 (for1)	Dimensions
Post submission		
Publications	z-score of total publications per year after submission by field	Dimensions
Citations	z-score of ln 1 + total of citations per year after submission by field	Dimensions
Other publication outcomes	Post submission: average relative citation ratio, average relative field ratio, average Altmetrics	Dimensions
Research income – post submission	'income secured yearly in £', 'income secured as PI in £'	Dimensions
Other early career awards	Whether unsuccessful MRC applicants received an early career award from another body	LinkedIn, Google

Table S2. Post estimation test for covariate balance

	χ^2	**p-value**
Total sample	10.002	0.694
Female only	6.287	0.901
Male only	7.769	0.803
