# Supplementary material for: Impact of early career medical research funding on subsequent publication outcomes and research income for female and male applicants in the United Kingdom
Source: Nat Commun. 2026 Jun 23;17:7909. doi: 10.1038/s41467-026-73009-z (PMC13442836; doi:10.1038/s41467-026-73009-z)
Supplement: Supplementary file 2 [file 41467_2026_73009_MOESM2_ESM.pdf]

nature portfolio

Corresponding author(s): Dr Charitini Stavropoulou

Last updated by author(s): Sep 30, 2025

Reporting Summary

Nature Portfolio wishes to improve the reproducibility of the work that we publish. This form provides structure for consistency and transparency in reporting. For further information on Nature Portfolio policies, see our Editorial Policies and the Editorial Policy Checklist.

Statistics

For all statistical analyses, confirm that the following items are present in the figure legend, table legend, main text, or Methods section.

n/a	Confirmed	
☐	☒	The exact sample size (n) for each experimental group/condition, given as a discrete number and unit of measurement
☐	☒	A statement on whether measurements were taken from distinct samples or whether the same sample was measured repeatedly
☐	☒	The statistical test(s) used AND whether they are one- or two-sided<br>*Only common tests should be described solely by name; describe more complex techniques in the Methods section.*
☐	☒	A description of all covariates tested
☐	☒	A description of any assumptions or corrections, such as tests of normality and adjustment for multiple comparisons
☐	☒	A full description of the statistical parameters including central tendency (e.g. means) or other basic estimates (e.g. regression coefficient) AND variation (e.g. standard deviation) or associated estimates of uncertainty (e.g. confidence intervals)
☐	☒	For null hypothesis testing, the test statistic (e.g. F, t, r) with confidence intervals, effect sizes, degrees of freedom and P value noted<br>*Give P values as exact values whenever suitable.*
☒	☐	For Bayesian analysis, information on the choice of priors and Markov chain Monte Carlo settings
☒	☐	For hierarchical and complex designs, identification of the appropriate level for tests and full reporting of outcomes
☐	☒	Estimates of effect sizes (e.g. Cohen's d, Pearson's r), indicating how they were calculated

Our web collection on statistics for biologists contains articles on many of the points above.

Software and code

Policy information about availability of computer code

Data collection	We match three databases: 1) the MRC's own records for key baseline characteristics (SIEBEL); 2) Dimensions, a database compiling research information of individual researchers, on research outcomes for research income and publication outcomes; and 3) manually identified information from LinkedIn and Google for career progression
Data analysis	The code used to analyse the data is available here: https://codeocean.com/capsule/3410317/tree

For manuscripts utilizing custom algorithms or software that are central to the research but not yet described in published literature, software must be made available to editors and reviewers. We strongly encourage code deposition in a community repository (e.g. GitHub). See the Nature Portfolio guidelines for submitting code & software for further information.

Data

Policy information about availability of data

All manuscripts must include a data availability statement. This statement should provide the following information, where applicable:

- Accession codes, unique identifiers, or web links for publicly available datasets
- A description of any restrictions on data availability
- For clinical datasets or third party data, please ensure that the statement adheres to our policy

This paper makes use of restricted data from UK Research and Innovation (UKRI), protected by the UK Data Protection Act 2018. This data was accessed by Dr Stavropoulou as part of a collaborative research project with UKRI. UKRI makes unrestricted data on its funding available via https://gtr.ukri.org/. Dimensions data was obtained from https://www.dimensions.ai. We manually searched LinkedIn dataset and Google engines.

A minimum dataset, created using the datasets above and removing any identifiable information is available here: https://codeocean.com/capsule/3410317/tree

Research involving human participants, their data, or biological material

Policy information about studies with human participants or human data. See also policy information about sex, gender (identity/presentation), and sexual orientation and race, ethnicity and racism.

Reporting on sex and gender	Our study uses self-reported information on the applicant's sex which participants were requested to submit at the application stage. Our full sample included 846 female and 1154 male applicants. Our analysis is done first for the full sample, followed by subgroup analysis for female and male applicants separately.
Reporting on race, ethnicity, or other socially relevant groupings	Our study uses self-reported information on ethnic background which participants were requested to submit at the application stage. The original classification included: Asian, Black, Mixed and White ethnic background. Due to small numbers in some categories, we conducted our analysis using two categories: white and non-white applicants. Our full sample included 1,596 white and 329 non-white applicants.
Population characteristics	In addition to gender and ethnicity described above, we also report on participants' age in years, as self-reported at the time they applied for funding. In our full sample, the average age of the study participants is 36.69 years.
Recruitment	Study participants include every individual researcher who applied for an MRC early career fellowship or award between 2006 and 2016.
Ethics oversight	The study received ethical approval from the School of Health Sciences (SHS) at City, University of London Ethics Committee (ETH2021-1239).

Note that full information on the approval of the study protocol must also be provided in the manuscript.

Field-specific reporting

Please select the one below that is the best fit for your research. If you are not sure, read the appropriate sections before making your selection.

☐ Life sciences ☒ Behavioural & social sciences ☐ Ecological, evolutionary & environmental sciences

For a reference copy of the document with all sections, see nature.com/documents/nr-reporting-summary-flat.pdf

Behavioural & social sciences study design

All studies must disclose on these points even when the disclosure is negative.

Study description	Our study is predominantly quantitative in nature and uses a counterfactual analysis. To understand the main findings of our analysis, we also conducted in-depth interviews with female applicants.
Research sample	Our sample consists of every individual researcher who applied for an early career fellowship or award from the Medical Research Council in the UK between 2006 and 2016. For the purposes of the main analysis, we focus only on those applicants who were assessed at the board level. We match three databases: 1) the MRC's own records for key baseline characteristics (SIEBEL); 2) Dimensions, a database compiling research information of individual researchers, on research outcomes for research income and publication outcomes; and 3) manually identified information from LinkedIn and Google for career progression.
Sampling strategy	As explained above, our sample consists of every individual researcher who applied for an MRC early career fellowship or award between 2006 and 2016. For the qualitative part of our study, we used purposive sampling.
Data collection	The MRC collects data from each applicant at the time of submission for an award or fellowship. Data were analysed in compliance with the MRC's data sharing policies.
Timing	Our sample consists of every individual researcher who applied for an early career fellowship or award from the Medical Research Council in the UK between 2006 and 2016. This allowed time post submission (at least 5 years) for the impact of the award to be observed.<br>Individual interviews were conducted between April 2021 and November 2021.
Data exclusions	We exclude applicants who appear as unsuccessful in our dataset, but secured an early career award from another funding body, such as the Wellcome Trust or the National Institute for Health and Care Research (NIHR), as these are competing awards to the ones we analyse in our study.
Non-participation	Non-participation is not applicable in our study.
Randomization	Study participants were classified as successful or unsuccessful if they had secured an MRC fellowship/award or not. Securing a grant is subject to a number of confounding factors, for which our counterfactual analysis (propensity score weighting) accounts for.

Reporting for specific materials, systems and methods

We require information from authors about some types of materials, experimental systems and methods used in many studies. Here, indicate whether each material, system or method listed is relevant to your study. If you are not sure if a list item applies to your research, read the appropriate section before selecting a response.

Materials & experimental systems

n/a	Involved in the study
☒	☐ Antibodies
☒	☐ Eukaryotic cell lines
☒	☐ Palaeontology and archaeology
☒	☐ Animals and other organisms
☒	☐ Clinical data
☒	☐ Dual use research of concern
☒	☐ Plants

Methods

n/a	Involved in the study
☒	☐ ChIP-seq
☒	☐ Flow cytometry
☒	☐ MRI-based neuroimaging

Plants

Seed stocks	*Report on the source of all seed stocks or other plant material used. If applicable, state the seed stock centre and catalogue number. If plant specimens were collected from the field, describe the collection location, date and sampling procedures.*
Novel plant genotypes	*Describe the methods by which all novel plant genotypes were produced. This includes those generated by transgenic approaches, gene editing, chemical/radiation-based mutagenesis and hybridization. For transgenic lines, describe the transformation method, the number of independent lines analyzed and the generation upon which experiments were performed. For gene-edited lines, describe the editor used, the endogenous sequence targeted for editing, the targeting guide RNA sequence (if applicable) and how the editor was applied.*
Authentication	*Describe any authentication procedures for each seed stock used or novel genotype generated. Describe any experiments used to assess the effect of a mutation and, where applicable, how potential secondary effects (e.g. second site T-DNA insertions, mosiacism, off-target gene editing) were examined.*
