# Supplementary material for: Impact of early career medical research funding on subsequent publication outcomes and research income for female and male applicants in the United Kingdom
Source: Nat Commun. 2026 Jun 23;17:7909. doi: 10.1038/s41467-026-73009-z (PMC13442836; doi:10.1038/s41467-026-73009-z)
Supplement: Supplementary file 3 [file 41467_2026_73009_MOESM3_ESM.pdf]

nature portfolio

Peer Review File

Impact of early career medical research funding on subsequent career outcomes for female and male applicants the UK

Corresponding Author: Dr Charitini Stavropoulou

This file contains all reviewer reports in order by version, followed by all author rebuttals in order by version.

Version 0:

Reviewer comments:

Reviewer #1

(Remarks to the Author)
Very interesting and competent paper. I recommend acceptance. They have an interesting question: the impact of funding on different demographic groups (gender in this case). The authors do a very nice job with an unusually rich and useful dataset. The econometrics is well designed and appropriate . The writing is clear and well put together

I would recommend a bit more clarification and motivation, as per the below
1. More motivation would be good - some discussion about the value for funders; discussion about different options for funders and a sense of the ROI. Tie in to other types of schemes - early career schemes are common in many other countries, so their results are likely to be of international interest
3. Can we learn more about the early career scheme? Are they awards to single researchers? Are they all the sane amount?
4. It would be helpful to have a little bit more motivation about the data as well - a lot of data is presented very early on without any motivation in Table 3. What's the model and the hypothesis? They need to spell out different measures of success - retention in academia (and why that might be success); publication, citations; and then the control variables - they are all muddled together in table 3. A discussion of what's missing would be helpful.
5. It'd also be helpful to have a discussion about robustness checks. Dimensions is a good dataset, and much more reliable than Scopus and Web of Science, but some discussion about the plusses and minusses would be good.
6. Dimensions uses multiple fields of research - which ones in particular?
7. ANy structural changes in the funding scheme between 2006 and 2016?

8. The use of propensity score matching should be played up a bit. It addresses the better at the threshold issue that Card et al. hypothesize. https://www.pnas.org/doi/full/10.1073/pnas.2212421120

9. Be good to have some interpretations as well. For x amount of $ you retain y more women..
10. The authors mention some qualitative analysis with 24 recipients. It'd be great to get their take on why the results are different for men and women. Is it a stronger signal for women than men? Is it that women are more productive with their time?
11. The paper ends with a bit of a whimper. What are the policy implications? Give all the Early Research awards to women? Surely not. Talk a bit about a future agenda

Reviewer #2

(Remarks to the Author)
Measuring the impact of research funding is hard, because the funders' decisions to fund research applications are bound to be based on the "quality" of the research and of the researcher, things that inevitably also affect the researchers' later success. In other words, we cannot conclude that the difference between the postsubmission - presubmission research (eg publications) outcomes of grantees and the postsubmission - presubmission outcomes of non-grantees is due to receiving the grants. It can simply be of selection bias: those who got the grant were higher quality and would have improved more with or without the grant.

If we add gender to the mix, measuring the impact of funding is even more difficult, since there may be gender differences in both the "quality" of the research and researcher AND gender differences in how the grants are evaluated, both which may

affect researchers' later success. In other words, there may be different selection bias for men and women.

Some of the differences in measurable characteristics of the funded and unfunded researchers (pre-submission) are seen in the paper's Table 3. But there no doubt are many other differences in other pre-submission characteristics of the researchers and of the proposed research besides these 12 characteristics, and many of them are not easily measurable (e.g. ambition, networks, novelty of project). If so, there will be selection bias even for those with identical levels of these 12 characteristics.

All this tells us that we cannot simply compare post-funding-decision productivity/citation etc. between those who were funded and those who were and say that they are the "effect of the funding."

The authors do acknowledge this in the methods/empirical strategy section (although not before) and think they have solved this problem. (Since solving the selection problem is so key to the paper's validity, they this should mention their approach much earlier in the paper.)

They say, "We use propensity score weighting for our analysis. The analysis accounts for differences observed at the baseline (submission year) between the treatment (successful) and control group (unsuccessful) and by balancing these differences, it allows for the differences in outcomes observed in the future can be attributed to the treatment (grant received). For the analysis to be valid, "a number of assumptions need to hold. First, unconfoundness or conditional exchangeability requires that all variables that affect the likelihood of receiving a grant are observed. Although this assumption cannot be tested empirically, we have rich information on pre-treatment outcomes suggesting that the assumption that all relevant variables are observed may be a reasonable approximation."

The intuition behind propensity score weighting as a solution for selection bias is that it makes weighted samples where the probability (propensity) of being treated – i.e. of receiving the award – is the same for the weighted samples of funded and unfunded applicants.

To know whether their propensity score weighting solved the selection bias or not, we need to know much more about what they did and what the results were. Particularly:
• First, I assume that this matching based on all 12 of the variables in Table 3 (such as age), including gender. This needs to be clarified.
• Second (and more importantly), we need to know how successful the propensity score weighting was. Once they have the weighted samples, the authors need to calculate the probability of being funded and see if it is the same for the weighted samples of those who were funded and those who weren't. Of course, there will some random difference, but it should be a very small percentage. (And, also, once they have the weighted samples, the average of the X's age etc of the two weighted samples should be around the same.) If the probabilities of being funded are clearly different for grantees' and non-grantees' weighted sample, then propensity score weighting did NOT solve their selection problems; in other words, these 12 variables alone were not adequate to predict who will be funded. (My guess is that the probabilities will not be the same. After all, if funders could predict who should be funded based only on these individual factors, why bother with a huge application and review process? These 12 variables don't even include anything about the research itself!)
• Third, not only does the probability of being funded have to be (approximately) the same for the weighted samples of those who were funded and those who weren't, but this also need to be true for men and women separately. After all, the impact of age, publications etc. on the likelihood of being funded, and/or the criteria for being funded, might very well be different for men and women. As a result, the authors need to do separate propensity score weighting on the male and female samples, and show that the funded and nonfunded weighted samples of men have (approx.) the same probability of being funded, and similarly for women.

I personally – and readers in general – cannot know whether they have solved the selection problem until these questions are answered and indicate that the propensity score weighting did (mostly) solve the selection problem. I doubt they will. If they don't, the paper does not measure the effect of funding on men or women.

Reviewer #3

(Remarks to the Author)
This paper examines gender differences in the impact of funding on subsequent career outcomes for Early Career Researchers (ECR). The paper finds that female grant recipients are more likely to be cited than unsuccessful female applicants. Female recipients also benefit more than male recipients. Using interviews, the authors find that female applicants benefited from maternity leave benefits and leadership and mentoring programs. This is a well-written paper that has an interesting set of results. Here I provide comments designed to improve the contribution of the paper.

First, the analysis is focused only on those applicants whose proposals are not triaged. In Table 1, it would be useful to report the overall success rates of the full sample and the analysis sample. I calculated that it was an 18% success rate for females and a 22% success rates for males for the full sample. It would be helpful to see the same for the analysis sample.

It would be good to see the gender breakdown and success rates in Table 2.

Should the second panel of Table 5 be Male Only?

Version 1:

Reviewer comments:

Reviewer #1

(Remarks to the Author)
The authors have done a good job of responding to my comments. I particularly like the additional analysis of the quality of the propensity score matching approach. It's hard to explain the technique for a more general purpose publication like Nature Communications, but they manage to do so. The point about the importance of influence is a good one - going beyond the mechanistic measures of publications

Reviewer #2

(Remarks to the Author)
The authors have done a good job of allaying my concerns and I now am willing to recommend acceptance.

Reviewer #4

(Remarks to the Author)
The paper provides interesting insight on the medium-term consequences of receiving vs not receiving early-career funding for early career researchers. It is well written and it seems to me that the authors have ticked all the right boxes in terms of statistical/robustness checks in their analysis. I have two major concerns though:

- the section devoted to qualitative interviews offers no detail whatsoever on how said interviews were conducted. The authors point to a different publication of theirs, but I believe that the present study should be self-sufficient and provide enough information on how the interviews were carried out and how the pool of interviewees was formed.

- more importantly, I think that the paper's major claim ("women benefit from early career research funding more than men") is simply unsustainable given the many limitations of the study in terms of data sample and size. Let us not lose sight of the fact that the results presented in the paper refer to (1) ~850 early career researchers, (2) based in the UK, (3) working in Medicine-adjacent fields, (4) between 2006 and 2016. I am by no means criticizing these limitations, as I am very well aware of how difficult it is to find the kind of data that the authors used in their study. At the same time, I don't think it is fair to extrapolate the findings obtained from such a small and specific sample of ECRs into the bold and highly generalistic claim in the paper's title

Version 2:

Reviewer comments:

Reviewer #4

(Remarks to the Author)

(Remarks on code availability)

Open Access This Peer Review File is licensed under a Creative Commons Attribution 4.0 International License, which permits use, sharing, adaptation, distribution and reproduction in any medium or format, as long as you give appropriate credit to the original author(s) and the source, provide a link to the Creative Commons license, and indicate if changes were made.
In cases where reviewers are anonymous, credit should be given to 'Anonymous Referee' and the source.
The images or other third party material in this Peer Review File are included in the article's Creative Commons license, unless indicated otherwise in a credit line to the material. If material is not included in the article's Creative Commons license and your intended use is not permitted by statutory regulation or exceeds the permitted use, you will need to obtain permission directly from the copyright holder.
To view a copy of this license, visit https://creativecommons.org/licenses/by/4.0/

<u>Response to reviewers' comments</u>

Reviewers' comments:

Reviewer #1 (Remarks to the Author):

Very interesting and competent paper. I recommend acceptance. They have an interesting question: the impact of funding on different demographic groups (gender in this case). The authors do a very nice job with an unusually rich and useful dataset. The econometrics is well designed and appropriate. The writing is clear and well put together.

We would like to thank the reviewer for their positive and constructive feedback. Below we address each point the reviewer has raised in more detail. Throughout our revisions we are mindful of the journal's word limit and we have tried to be as concise as possible when adding new text in the manuscript.

I would recommend a bit more clarification and motivation, as per the below
1. More motivation would be good - some discussion about the value for funders; discussion about different options for funders and a sense of the ROI. Tie into other types of schemes - early career schemes are common in many other countries, so their results are likely to be of international interest

In the introduction of the revised document, we talk more about the role of funders in supporting ECRs, how they have created schemes specifically for them as well as the role they play in dealing with the bigger issue of precarity in research. We have not come across any study that considers ROI for ECRs.

We absolutely agree with the reviewer that this is an issue of international interest and we provide examples of similar schemes around the world and cite the OECD report on precarity among ECRs globally.

3. Can we learn more about the early career scheme? Are they awards to single researchers? Are they all the same amount?

We have added more information regarding the three schemes that are considered in our study in the methods section. We are mindful of the world limit, but we would be happy to include additional information if the reviewer believes it is needed.

4. It would be helpful to have a little bit more motivation about the data as well - a lot of data is presented very early on without any motivation in Table 3. What's the model and the hypothesis? They need to spell out different measures of success - retention in academia (and why that might be success); publication, citations; and then the control variables - they are all muddled together in table 3. A discussion of what's missing would be helpful.

This is a fair point. Details about the various variables are presented in the methods section which appears at the end of the manuscript as per the journal's guidelines. We have now added text in the introduction of the paper to present the model and its hypotheses and the variables used both at the baseline and as outcome measures

(retention is not included as we only hold information on where applicants were in 2021, and this does not tell us the full story). We explained how our decision was driven by previous literature and shaped up by the data available to us. We also added text in the discussion on what other variables would have been useful to have, such as information on tenure and promotions.

5. It'd also be helpful to have a discussion about robustness checks. Dimensions is a good dataset, and much more reliable than Scopus and Web of Science, but some discussion about the plusses and minusses would be good.

We have added a discussion on Dimensions vs Scopus and Web of Science and the choice of Dimensions as the key database. We didn't run any robustness checks to compare the three, but there is evidence that they seem to perform in rather similar ways: [*https://www.sciencedirect.com/science/article/abs/pii/S175115771830066X*](https://www.sciencedirect.com/science/article/abs/pii/S175115771830066X)

6. Dimensions uses multiple fields of research - which ones in particular?

Indeed, Dimensions uses multiple fields of research. In our analysis, we used field of research level 1 (for1). Please see Table 7.

7. Any structural changes in the funding scheme between 2006 and 2016?

There have not been any structural changes in the funding scheme that affect our cohort.

8. The use of propensity score matching should be played up a bit. It addresses the better at the threshold issue that Card et al. hypothesize. https://www.pnas.org/doi/full/10.1073/pnas.2212421120

Thank you for finding merit in the methods we use. As every method, it has pros and cons and we now provide a more extensive discussion of our choice of model in the methods section.

9. Be good to have some interpretations as well. For x amount of $ you retain y more women.

This is an interesting point, but retention was not one of our outcome measures, as mentioned above.

10. The authors mention some qualitative analysis with 24 recipients. It'd be great to get their take on why the results are different for men and women. Is it a stronger signal for women than men? Is it that women are more productive with their time?

We provide more information about the qualitative analysis in the discussion session. The qualitative study showed significant gender differences, with 14 out of 24 female interviewees having taken a maternity leave during their award, while no male interviewee mentioned a career break. Maternity policies, mentorship and leadership schemes specific to women, may partly explain our results; women who get an award benefit more than women who did not get it because the awards come with

supportive policies that help them navigate better challenges that women face in research. These policies are probably less relevant for men.

11. The paper ends with a bit of a whimper. What are the policy implications? Give all the Early Research awards to women? Surely not. Talk a bit about a future agenda

Indeed, we are not suggesting that more funding should be offered to women. Although more work is needed to understand the mechanisms that may support women in doing better, and we suggest that future work could focus on that, our work seems to support the idea that post award support for women matters and may result in successful female applicants doing better than unsuccessful one.

Reviewer #2 (Remarks to the Author):

Measuring the impact of research funding is hard, because the funders' decisions to fund research applications are bound to be based on the "quality" of the research and of the researcher, things that inevitably also affect the researchers' later success. In other words, we cannot conclude that the difference between the postsubmission - presubmission research (eg publications) outcomes of grantees and the postsubmission - presubmission outcomes of non-grantees is due to receiving the grants. It can simply be of selection bias: those who got the grant were higher quality and would have improved more with or without the grant.

The reviewer is right in that measuring the impact of research funding is hard. This should not diminish the efforts to attempt an understanding of the phenomenon. Our paper contributes to a very scarce literature on the impact of funding among early career researchers and is the first one to try to explore who benefits more from it.

If we add gender to the mix, measuring the impact of funding is even more difficult, since there may be gender differences in both the "quality" of the research and researcher AND gender differences in how the grants are evaluated, both which may affect researchers' later success. In other words, there may be different selection bias for men and women.

Our study is based on the hypothesis that there may indeed be differences among men and women. Which is why we first conduct the analysis using the full sample and then we do two subgroup analyses for male and female applicants separately.

Some of the differences in measurable characteristics of the funded and unfunded researchers (pre-submission) are seen in the paper's Table 3. But there no doubt are many other differences in other pre-submission characteristics of the researchers and of the proposed research besides these 12 characteristics, and many of them are not easily measurable (e.g. ambition, networks, novelty of project). If so, there will be selection bias even for those with identical levels of these 12 characteristics.

All this tells us that we cannot simply compare post-funding-decision productivity/citation etc. between those who were funded and those who were and say that they are the "effect of the funding."

The authors do acknowledge this in the methods/empirical strategy section (although not before) and think they have solved this problem. (Since solving the selection problem is so key to the paper's validity, they this should mention their approach much earlier in the paper.)

Indeed, we acknowledge this in the methods section, which, in the structure that Nature Communications has, it appears after the results. As every model, ours is based on assumptions, it has strengths and limitations which we try to address and acknowledge. We provide more explanation on the choice of our model in the methods section.

They say, "We use propensity score weighting for our analysis. The analysis accounts for differences observed at the baseline (submission year) between the treatment (successful) and control group (unsuccessful) and by balancing these differences, it allows for the differences in outcomes observed in the future can be attributed to the treatment (grant received).
For the analysis to be valid, "a number of assumptions need to hold. First, unconfoundness or conditional exchangeability requires that all variables that affect the likelihood of receiving a grant are observed. Although this assumption cannot be tested empirically, we have rich information on pre-treatment outcomes suggesting that the assumption that all relevant variables are observed may be a reasonable approximation."

The intuition behind propensity score weighting as a solution for selection bias is that it makes weighted samples where the probability (propensity) of being treated – i.e. of receiving the award – is the same for the weighted samples of funded and unfunded applicants.

To know whether their propensity score weighting solved the selection bias or not, we need to know much more about what they did and what the results were. Particularly:
• First, I assume that this matching based on all 12 of the variables in Table 3 (such as age), including gender. This needs to be clarified.

Indeed, all variables on Table 3 (now Table 4) are the observable characteristics that our model accounts for. Gender is naturally omitted when the analysis focuses on male and female applicants separately.

• Second (and more importantly), we need to know how successful the propensity score weighting was. Once they have the weighted samples, the authors need to calculate the probability of being funded and see if it is the same for the weighted samples of those who were funded and those who weren't. Of course, there will some random difference, but it should be a very small percentage. (And, also, once they have the weighted samples, the average of the X's age etc of the two weighted samples should be around the same.) If the probabilities of being funded are clearly different for grantees' and non-grantees' weighted sample, then propensity score weighting did NOT solve their selection problems; in other words, these 12 variables alone were not adequate to predict who will be funded. (My guess is that the probabilities will not be the same. After all, if funders could predict who should be

funded based only on these individual factors, why bother with a huge application and review process? These 12 variables don't even include anything about the research itself!)

The weighting of probabilities of success is a built-in mechanism in the model. In other words, it is the purpose of propensity score to balance the differences between the treatment and control group. Nevertheless, to reassure you that the model was successful in doing so, we provide two new tables as seen below, responding directly to the concerns you raise.

Table X1 below shows that the probability of winning an award for the treatment group (successful candidates) is 56.64% while for the control (unsuccessful candidates) is 47.05%. After weighting the sample using the propensity score we have calculated, the two groups have almost identical probabilities of receiving an award; 51.96% vs 51.86% respectively. This holds for the total sample, but also for male and female separately.

***Table X1:** Probability of receiving an award before and after propensity score weighting*

	Unweighted		*Weighted*	
Total				
	Mean	*Std. dev.*	*Mean*	*Std. dev.*
Successful	*0.5664*	*0.1452*	*0.5195*	*0.1563*
Unsuccessful	*0.4705*	*0.1495*	*0.5186*	*0.1524*
Male				
	Mean	*Std. dev.*	*Mean*	*Std. dev.*
Successful	*0.5922*	*0.1597*	*0.5322*	*0.1792*
Unsuccessful	*0.4675*	*0.1701*	*0.5322*	*0.1742*
Female				
	Mean	*Std. dev.*	*Mean*	*Std. dev.*
Successful	*0.5467*	*0.1475*	*0.5041*	*0.1472*
Unsuccessful	*0.4532*	*0.1447*	*0.5050*	*0.1607*

Table X2 responds to concerns that differences in the characteristics of the two samples should be balanced after weighting the sample. The table illustrates that, wherever there were observable differences (age, previous income, past applications and clinical background), these differences do no longer exist when applying propensity score weighting. This is true both for the total sample and for male and female candidates separately. We have replaced Table 3 of the original submission with this table (now Table 4).

***Table X2**: Differences in observable characteristics between successful and unsuccessful applicants before and after propensity score weight adjustment (at board)*

Successful vs. unsuccessful applicants						
	Total		*Male*		*Female*	
	Unweighted	*Weighted*	*Unweighted*	*Weighted*	*Unweighted*	*Weighted*

	p-value	*p-value*	*p-value*	*p-value*	*p-value*	*p-value*
Age	***<0.001***	*0.923*	***<0.001***	*0.975*	***<0.001***	*0.833*
Gender	*0.473*	*0.957*	*N/A*	*N/A*	*N/A*	*N/A*
Ethnicity	*0.203*	*0.799*	*0.255*	*0.851*	*0.429*	*0.866*
Institution in London/Oxbridge	*0.484*	*0.873*	*0.694*	*0.982*	*0.583*	*0.952*
Publications (standardised)	*0.598*	*0.801*	*0.425*	*0.926*	*0.061*	*0.590*
Citations (standardised)	*0.133*	*0.789*	*0.671*	*0.981*	*0.092*	*0.725*
RCR (standardised)	*0.083*	*0.836*	*0.222*	*0.850*	*0.277*	*0.961*
FCR (standardised)	*0.079*	*0.809*	*0.310*	*0.788*	*0.171*	*0.935*
Altmetrics (standardised)	*0.263*	*0.602*	*0.380*	*0.702*	*0.360*	*0.533*
Previous Income (binary)	***<0.001***	*0.970*	***<0.001***	*0.873*	***<0.001***	*0.937*
Past applications (binary)	***<0.001***	*0.932*	***<0.001***	*0.845*	***0.041***	*0.805*
Clinical background	***0.011***	*0.825*	***0.117***	*0.787*	***0.011***	*0.883*

Together the two tables illustrate clearly that the concerns that the model does not resolve the selection problem are misplaced.

• Third, not only does the probability of being funded have to be (approximately) the same for the weighted samples of those who were funded and those who weren't, but this also need to be true for men and women separately. After all, the impact of age, publications etc. on the likelihood of being funded, and/or the criteria for being funded, might very well be different for men and women. As a result, the authors need to do separate propensity score weighting on the male and female samples, and show that the funded and nonfunded weighted samples of men have (approx.) the same probability of being funded, and similarly for women.

Indeed, as Tables X1 and X2 above illustrate, the weighted probabilities are very similar both for female and male candidates separately.

I personally – and readers in general – cannot know whether they have solved the selection problem until these questions are answered and indicate that the propensity score weighting did (mostly) solve the selection problem. I doubt they will. If they don't, the paper does not measure the effect of funding on men or women.

Together the two tables provide further, stronger evidence that the model successfully resolves the selection problem.

Reviewer #3 (Remarks to the Author):

This paper examines gender differences in the impact of funding on subsequent career outcomes for Early Career Researchers (ECR). The paper finds that female grant recipients are more likely to be cited than unsuccessful female applicants. Female recipients also benefit more than male recipients. Using interviews, the authors find that female applicants benefited from maternity leave benefits and leadership and mentoring programs. This is a well-written paper that has an interesting set of results. Here I provide comments designed to improve the contribution of the paper.

Thank you for your positive comments and your constructive feedback. Below we reply to each point separately.

- First, the analysis is focused only on those applicants whose proposals are not triaged. In Table 1, it would be useful to report the overall success rates of the full sample and the analysis sample. I calculated that it was an 18% success rate for females and a 22% success rates for males for the full sample. It would be helpful to see the same for the analysis sample.

This is a valid point. To avoid confusion, given that Table 1 reports on the full sample, we have created a new table with success rates by gender separately for the full sample and the analysis sample (those who reached the board level). This is now Table 3 in the revised document. We also make a note, that differences in success rates between men and women are not significant after once controls for a number of variables including age, ethnicity, institution, past publications, income and clinical background. We would be happy to present the logit regressions we run that justify this point, but we are mindful of the word limit and extra tables. We could discuss with the editor.

- It would be good to see the gender breakdown and success rates in Table 2.

As per our previous response, we created a separate table with the success rates by gender for the full and the analysis sample, which answers the reviewer’s point. Please see new Table 3 in the revised document.

- Should the second panel of Table 5 be Male Only?

Thank you for spotting the inconsistency. Indeed, it should be Male Only. We have made the change in the revised document.

Response to reviewer comments

Reviewer #1 (Remarks to the Author):

The authors have done a good job of responding to my comments. I particularly like the additional analysis of the quality of the propensity score matching approach. It's hard to explain the technique for a more general purpose publication like Nature Communications, but they manage to do so. The point about the importance of influence is a good one - going beyond the mechanistic measures of publications

We would like to thank the reviewer again for their comments on our work that we believe have improved our work. Thank you very much for finding merit in our revisions.

Reviewer #2 (Remarks to the Author):

The authors have done a good job of allaying my concerns and I now am willing to recommend acceptance.

Thank you very much for finding merit in our revisions. We appreciate the comments you provided in our work.

Reviewer #4 (Remarks to the Author):

The paper provides interesting insight on the medium-term consequences of receiving vs not receiving early-career funding for early career researchers. It is well written and it seems to me that the authors have ticked all the right boxes in terms of statistical/robustness checks in their analysis.

Thank you very much for your positive feedback on our work. Below we are trying to address your concerns.

I have two major concerns though:

- the section devoted to qualitative interviews offers no detail whatsoever on how said interviews were conducted. The authors point to a different publication of theirs, but I believe that the present study should be self-sufficient and provide enough information on how the interviews were carried out and how the pool of interviewees was formed.

> *We agree more information is needed to help the reader understand the qualitative study without reading the published paper. We have added a paragraph to explain how interviewees were chosen and how interviews were*

conducted. We are happy to provide more information if the reviewer feels it is needed.

- more importantly, I think that the paper's major claim ("women benefit from early career research funding more than men") is simply unsustainable given the many limitations of the study in terms of data sample and size. Let us not lose sight of the fact that the results presented in the paper refer to (1) ~850 early career researchers, (2) based in the UK, (3) working in Medicine-adjacent fields, (4) between 2006 and 2016. I am by no means criticizing these limitations, as I am very well aware of how difficult it is to find the kind of data that the authors used in their study. At the same time, I don't think it is fair to extrapolate the findings obtained from such a small and specific sample of ECRs into the bold and highly generalistic claim in the paper's title

This is a fair comment. We discuss as the main limitation of our study the fact that it concerns a small and specific sample of ECRs and that larger studies looking across fields would be helpful to consider the role of sex, but also gender and ethnicity when considering the effects of funding. What our study hopefully offers, is a shift of the debate on scientific funding from exploring what the effect on the quality and quantity of scientific outputs is, to also who is more likely to benefit from such support.

We have also changed the title to reflect on this and clarify our focus is on medical research only. We still think a reference to sex should be on the title, but hopefully this is now less bold than before.
